# Prevalence and genotypes of group A rotavirus among outpatient children under five years old with diarrhea in Beijing, China, 2011–2016

**DOI:** 10.1186/s12879-018-3411-3

**Published:** 2018-10-03

**Authors:** Yi Tian, Abrar Ahmad Chughtai, Zhiyong Gao, Hanqiu Yan, Yanwei Chen, Baiwei Liu, Da Huo, Lei Jia, Quanyi Wang, Chandini Raina MacIntyre

**Affiliations:** 1 Institute for Infectious Disease and Endemic Disease Control, Beijing Municipal Center for Disease Prevention and Control, Beijing, China; 2 Institute for Infectious Disease and Endemic Disease Control, Beijing Research Center for Preventive Medicine, Beijing, China; 3 School of Public Health and Community Medicine, University of New South Wales, Sydney, Australia; 4 College of Public Service and Community Solutions, and College of Health Solutions, Arizona State University, Phoenix, USA

**Keywords:** Diarrhea, Children under five years old, Group A rotavirus, Genotype

## Abstract

**Background:**

Rotavirus is a leading cause of severe diarrheal disease, and one of the common causes of death in children aged under five years old. The dominant epidemic strains may change in different years in the same area. In order to provide evidence for rotavirus epidemic control and inform vaccine development, we analyzed epidemiological patterns and genetic characteristics of rotavirus in Beijing during 2011–2016.

**Methods:**

Stool specimens of outpatient children under five years old were collected from three children’s hospitals on a weekly basis. Group A rotavirus antigens were detected using enzyme-linked immunosorbent assay (ELISA) kit. The partial VP4 genes and VP7 genes of rotavirus were both amplified and sequenced. Genotyping and phylogenetic analyses were performed. Logistic regression and Chi-square tests were performed to determine differences across age groups, districts and years in rotavirus prevalence and genotype distribution.

**Results:**

A total of 3668 stool specimens from children with acute diarrhea identified through hospital-based surveillance were collected from 2011 to 2016 in Beijing. A total of 762 (20.8%) specimens tested positive for rotavirus. The rotavirus-positive rate was highest among the 1–2 years old age group (29.0%, 310/1070). November, December and January were the highest rotavirus-positive rate months each year. G9 was the most common G genotype (64.4%, 461/716), and P [8] was the most common P genotype (87.0%, 623/716) among the 716 rotavirus-positive specimens. G9P [8], G3P [8] and G2P [4] were the most common strains. The rotavirus-positive rates of samples in 2012 and 2013 were higher than that in 2011, and the dominant genotype changed from G3P [8] to G9P [8] in 2012 and 2013. VP7 gene sequences of G9 strains in this study clustered into two main lineages. Most of the G9 strains exhibited the highest nucleotide similarity (99.1%~ 100.0%) to the strain found in Japan (MI1128). VP4 gene sequences of P [8] strains were almost P[8]b.

**Conclusions:**

Rotavirus accounted for more than one fifth of childhood diarrhea in Beijing during the study period. Targeted measures such as immunization with effective rotavirus vaccines should be carried out to reduce the morbidity and mortality due to rotavirus.

## Background

Infection with group A rotavirus is the main cause of severe diarrheal disease in children worldwide [1], and one of the common causes of death in children aged under 5 years old [2, 3]. According to World Health Organization (WHO), approximately 215,000 children aged under 5 years old died from rotavirus infection in 2013 [4]. In China, 40% of diarrhea-related hospitalizations and 30% of diarrhea-related outpatient visits among children younger than 5 years old from 1994 through 2014 were caused by group A rotavirus [5]. From 2003 to 2012, rotavirus infection contributed to 42% of diarrhea-associated death among Chinese children under 5 years old [6].

According to the sequence diversity of VP7 and VP4 genes, group A rotavirus can be divided into several G and P genotypes [7, 8]. So far, there have been 27 G genotypes and 37 P genotypes identified. Other new G and P genotypes are expected to be identified in the future [9–12]. G3P [8], G1P [8], G2P [4] and G4P [8] have been the four dominant strains from 1989 through 2004 globally [13–15]. Since 2004, G9P [8] was detected as one of the major epidemic strains, gradually increasing worldwide [16]. However, the major epidemic strains of group A rotavirus varies by countries and regions. Moreover, the major epidemic strains may change in different years in the same area [7, 17, 18]. In China, G1P [8] was the dominant strain from1983–2000 [19, 20]. The proportion of G3 increased dramatically from 2000 to 2007, and G3P [8] became the dominant strain [21–23]. In 2008, G9 was reported to be a highly prevalent genotype in Xinjiang in the northwest part of China like other countries [24–26].

There is no specific drug for the rotavirus diarrhea, but effective vaccines are available and can protect children from illness and death caused by rotavirus. In China, only the LLR (Lanzhou Institute of Biological Products, Lanzhou, China) vaccine is licensed for use, which is sourced from G10P [12] of sheep. However, this vaccine has not yet been included in the national immunization program, and there are no available clinical trial data so far. Further understanding the distribution of rotavirus strains may provide evidence for the use of vaccines in China. Previous data in China, usually based on a short-term observational studies, are limited. However, in this study we presented the results of 6-years of surveillance data to further elucidate the changing epidemiology, and thus inform targeted control measures and vaccine development.

## Methods

### Data source

Data were collected on children with acute diarrhea identified through hospital-based surveillance from 2011 to 2016 in Beijing. Acute diarrhea cases were defined as outpatient children aged under 5 years old who had diarrhea (3 or more loose stools within 24 h). Children who were not residents in Beijing were excluded. Sentinel hospital surveillance for rotavirus diarrhea among children under 5 years old was established in three children’s hospitals located in Xicheng, Chaoyang and Tongzhou districts respectively. Stool specimens and socio-demographic data were collected from children under 5 years old who were newly diagnosed with acute diarrhea at the first medical consultation. Fifteen specimens were collected every month from each sentinel hospital. Socio-demographic information includes district, surveillance month, age and sex. District centers for disease control (CDC) are responsible for sending specimens and information of patients to Beijing CDC each month.

### Laboratory testing

#### Detection of group A rotaviruses

Rotavirus antigen detection was performed by enzyme linked immunosorbent assay (ELISA) using ProSpecT Rotavirus Microplate Assay (Oxoid Ltd., Basingstoke Hants, UK), according to the manufacturer’s instructions.

### Viral RNA extraction

According to the manufacturer’s protocol, QIAamp Viral RNA Mini Kit (QIAGEN, Hilden, Germany) was used to extract Viral RNA from 140 μL of a 10% fecal suspension in phosphate-buffered saline. Extracted RNA was stored at − 20 °C until further use.

### Genotyping of group A rotaviruses

Rotavirus G and P genotyping were determined by multiplex semi-nested reverse transcription polymerase chain reaction (RT-PCR) [27, 28]. A total of 5 μL rotavirus RNA was mixed with 5 μL universal primers, primers VP7F/VP7R for G typing and primers VP4F/VP4R for P typing. The mixture was denatured at 98 °C for 5 min and cooled on ice for 5 min, then the one-step RT-PCR kit (QIAGEN, Hilden, Germany) was used to amplify VP7 and VP4 genes in a 50 μL reaction volume. RT-PCR was performed at 50 °C for 30 min and 95 °C for 15 min followed by 30 cycles of 94 °C for 30 s, 42 °C for 30 s, and 72 °C for 60 s. A final extension was run at 72 °C for 7 min.

The second-round PCR was performed using AmpliTaq Gold® 360 PCR Master Mix (Life Tech, Foster City, CA, USA). For G genotyping, primers aBT1, aCT2, G3, aDT4, aAT8, G9, G12 and VP7R were used, generating amplicons of 618 bp, 521 bp, 682 bp, 452 bp, 754 bp, 179 bp and 387 bp for G1, G2, G3, G4, G8, G9 and G12, respectively. For P genotyping, primers VP4F, 1 T-1D, 2 T-1, 3 T-1, 4 T-1, 5 T-1, and P [11] were used, generating amplicons of 362 bp, 146 bp, 224 bp, 270 bp, 462 bp and 191 bp for P [4], P [6], P [8], P [9], P [10] and P [11], respectively. PCR was performed at 95 °C for 10 min followed by 35 cycles of 95 °C for 30 s, 42 °C for 30 s, and 72 °C for 60 s; a final extension was run at 72 °C for 7 min. The PCR products were analyzed using a QIAxcel Advanced Instrument with a QIAxcel DNA Screening Kit (QIAGEN, Hilden, Germany).

### DNA sequencing and phylogenetic analysis

The first-round PCR products were purified and sequenced directly on an ABI 3730xl DNA Analyzer using a BigDye Terminator v3.1 Cycle Sequencing Kit (ABI, Austin, TX, USA). All sequences were prepared and aligned by BioEdit (version 7.0.9.0) with the Clustal W program. The phylogenetic tree was constructed using the neighbor-joining method with MEGA software (version 6.06) and bootstrap analysis was performed with 1000 replications. The nucleotide sequences used in this study were submitted in GenBank under accession numbers MH631063-MH631154, MH625726-MH625776 and MH625777-MH625915. The reference sequences used in plotting the phylogeny tree from the GenBank database are shown in Table 1.

**Table 1:** Reference sequences used in plotting the phylogeny tree of this study

Strains	Accession numbers	genotypes	Starains	Accession numbers	genotypes
MI1128	LC228408	G9	CNMC3	KT920808	P [8]
BJ-Q322	KF673479	G9	CK00095	JX027933	P [8]
VU12-13-101	KT919508	G9	UR14-26	LC105523	P [8]
MRC-DPRU1102	KJ753473	G9	X1302	KJ794102	P [8]
Km15064	KX033644	G9	H1301	KJ794103	P [8]
Hon-JK17–6-14	KU312101	G9	Z1557	KF372006	P [8]
BJ-Q532	KF673480	G9	WZ7	KU243615	P [8]
99-SP1542VP7	AB091753	G9	AC314	KT025876	P [8]
99-TK2082VP7	AB091755	G9	To14–18	LC105271	P [8]
JP13-3	AB176679	G9	SI-R13	KJ432798	P [8]
LL51695	KC242226	G9	D	EF672570	P [8]
WZ660	KU243678	G9	ISO42	DQ355958	P [8]
km15118	KX778607	G9	Nov10-N709	HQ537506	P [8]
MRC-DPRU5123	KP752521	G9	LY238-9	KU174038	P [4]
F45	AB180970	G9	N14-29	LC342710	P [4]
AU32	AB045372	G9	RMRC-11-03-0614	MF373697	P [4]
N12–55	LC380583	G1	O1157	JX156413	P [4]
N10–17	LC348885	G1	07–96 s-98	KY489875	P [4]
CK20027	KC443768	G2	WZ193	KU243633	P [4]
km15028	KX033592	G2			
VU12–13-27	MF167970	G2			
E3239	KF371889	G3			
E2432	KF371856	G3			
1RIZE2016	MF494811	G3			

### Statistical analysis

Socio-demographical data and laboratory data were double entered and checked. Data analyses were performed using SPSS (version 20.0). Univariate logistic regression analyses were conducted to identify factors predictive of rotavirus infection. Multivariate logistic regression was conducted to adjust for confounders. Chi-square test was used to compare the distribution of GP genotypes between different genders, age groups, living areas and years. All hypothesis tests were 2-tailed, and a probability of *P* < 0.05 was considered statistically significant.

## Results

### Patients with diarrhea

A total of 3668 stool specimens of children with acute diarrhea were collected from 3668 cases, including 2296 specimens from boys, 1363 specimens from girls, 9 cases of unknown gender. A total of 762 stool specimens tested positive for rotavirus with the positive rate of 20.8%.

**Fig. 1:**
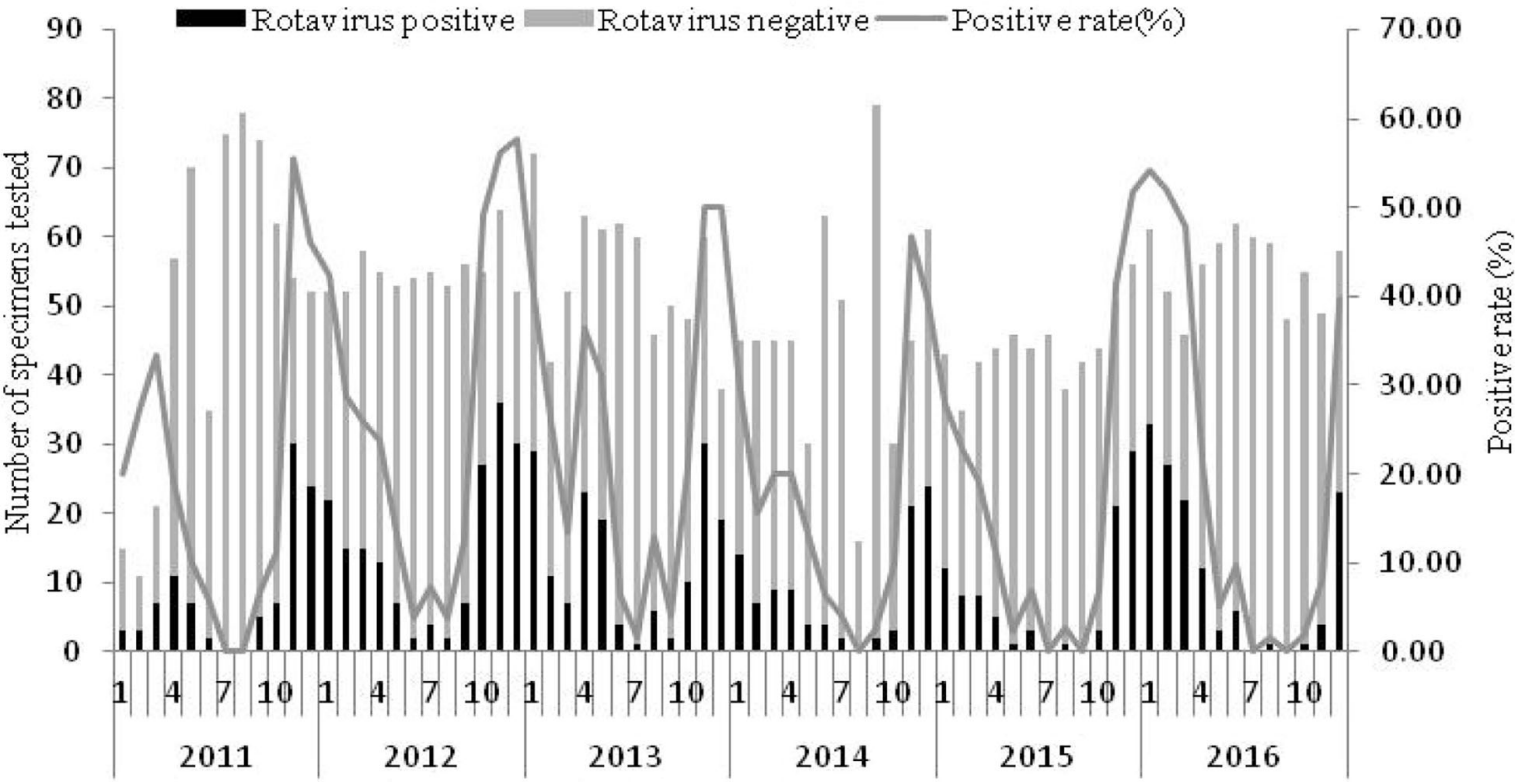
Monthly distribution of the number and positive rate of rotavirus among outpatient children under five years old with diarrhea in Beijing, China, 2011–2016

In 22 children, age was unknown. The rotavirus-positive rate in children aged less than 6 months was the lowest (9.0%). Children aged 1–2 years old had the highest rotavirus-positive rate (29.0%), followed by those aged 6–11 months old (24.0%), and children aged 2–3 years old (17.9%) (Table 2). Compared with children 0–5 months, age groups 6–11 months, 1–2 and 2–3 years old were at higher risk for rotavirus-positive. The rotavirus-positive rate in urban children was 23.9% (585/2480). Children who lived in rural areas were less likely to be rotavirus-positive (*OR* 0.57, 95% *CI* 0.47–0.68). From 2011 to 2016, the rotavirus-positive rates were 16.4% (99/604), 27.3% (180/659), 24.6% (161/654), 17.8% (99/555), 17.1% (91/531), and 19.8% (132/665), respectively each year. The rotavirus-positive rates in 2012 and 2013 were higher, being > 20%. Controlled for age and urban residence, rotavirus-positive rates were significantly different between different years (*P* < 0.001). The rotavirus-positive rates in 2012 and 2013 were higher, compared with 2011. Adjusted *OR* was 1.90 (95% *CI* 1.43–2.54) in 2012 and 1.60 (95% *CI* 1.19–2.15) in 2013. The largest number and highest rotavirus-positive rate of specimens were in November, December and the following January each epidemic season (Fig. 1).

**Table 2:** Demographic characteristics of the outpatient children under five years old with diarrhea in Beijing, China, 2011–2016

Demographicharacteristics	Total number	Number of rotavirus positive(%)	OR	95%*CI*	*P*-value
Gender^a^					
Male	2296	482 (21.0%)	1.00	Reference	
Female	1363	271 (19.9%)	0.93	0.79–1.10	0.422
Age, years^a^					
0–5 months	837	75 (9.0%)	1.00	Reference	
6–11 months	1179	283 (24.0%)	3.21	2.44–4.21	< 0.01
1-	1070	310 (29.0%)	4.14	3.16–5.44	< 0.01
2-	285	51 (17.9%)	2.21	1.51–3.25	< 0.01
3-	150	18 (12.0%)	1.39	0.80–2.39	0.242
4-	125	12 (9.6%)	1.08	0.57–2.05	0.816
Living area					
Urban	2480	585 (23.9%)	1.00	Reference	
Rural	1188	177 (14.9%)	0.57	0.47–0.68	< 0.01
Years					
2011	604	99 (16.4%)	1.00	Reference	
2012	659	180 (27.3%)	0.79	0.59–1.06	0.111
2013	654	161 (24.6%)	1.52	1.17–1.96	0.001
2014	555	99 (17.8%)	1,32	1.02–1.71	0.038
2015	531	91 (17.1%)	0.88	0.66–1.17	0.372
2016	665	132 (19.8%)	0.84	0.62–1.12	0.232

^a^There are missing data

Among 762 rotavirus positive specimens, 716 (94.0%) were genotyped successfully for G and P (Table 3). G9 was the most common G genotype, accounting for 64.4% (461/716). P [8] was the most common P genotype, accounting for 87.0% (623/716).

**Table 3:** The distribution of G and P genotypes of rotavirus positive specimens isolated from outpatient children under five years old with diarrhea in Beijing, China, 2011–2016

	Total number	P [4]	P [8]	P [9]	P Mix	UT
G1	35	0	35	0	0	0
G2	57	47	8	0	1	1
G3	68	1	64	0	3	0
G8	1	0	1	0	0	0
G9	461	5	446	0	4	6
G Mix	67	5	55	0	6	1
UT	27	0	14	1	1	11
Total	716	58	623	1	15	19

**Fig. 2:**
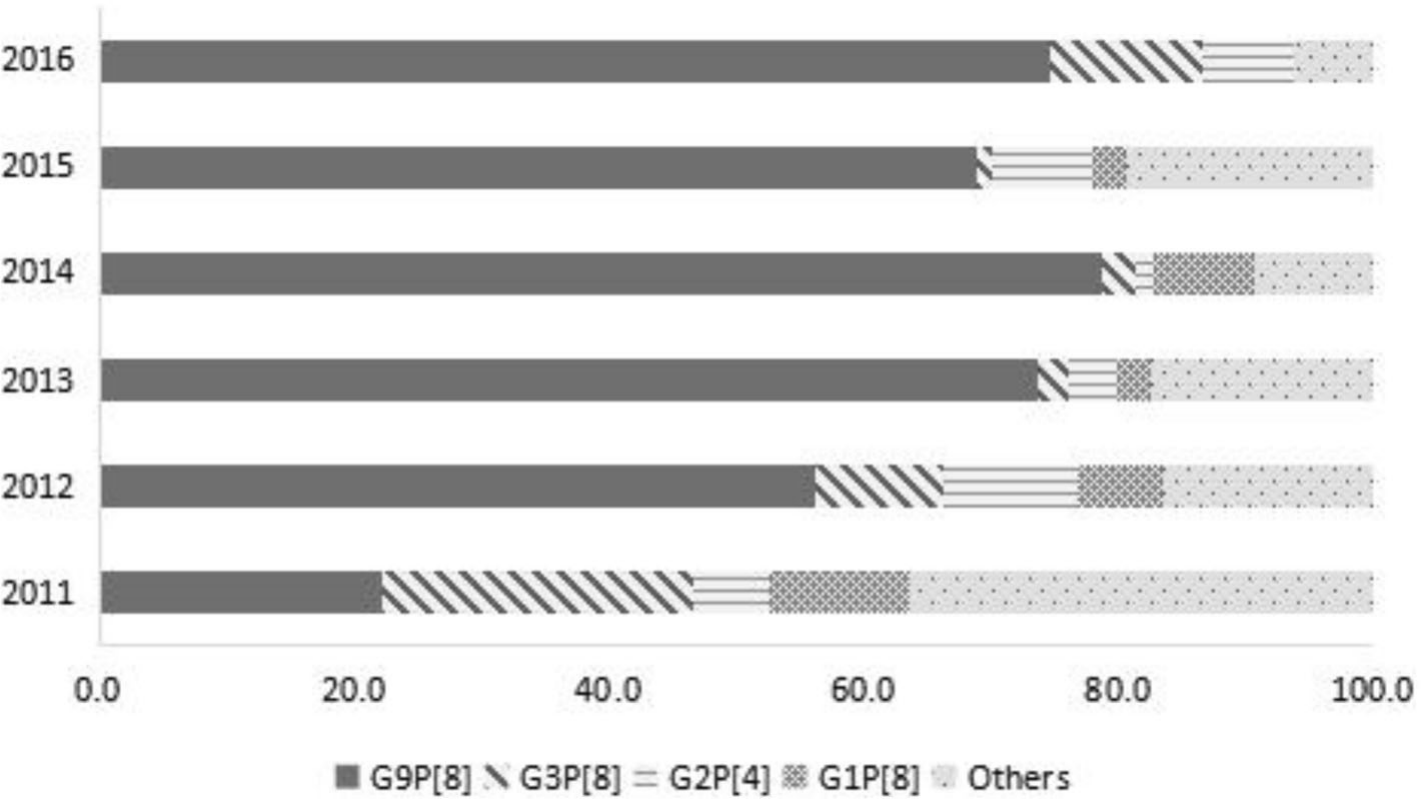
The change of dominant genotypes of rotavirus-positive specimens isolated from outpatient children under five years old with diarrhea in Beijing, China, 2011–2016

The most common GP combined genotype was G9P [8] (62.3%, 446/716), followed by G3P [8] and G2P [4]. G3P [8] and G9P [8] mixed accounted for 48.4% (46/95) of the others. There was no statistically significant difference in distribution of genotype by gender (*χ*^2^ = 4.492, *P* = 0.344), age group (*χ*^2^ = 15.036, *P* = 0.774) or living area (*χ*^2^ = 2.906, *P* = 0.574). In 2011, G3P [8] and G9P [8] were both the dominant genotypes, accounting for 24.2 (24/99) and 22.2% (22/99) of samples respectively. In 2012, G9P [8] became the dominant genotype, accounting for 56.1% (101/180) of samples. From 2013 to 2016, G9P [8] was the dominant genotype, accounting for 70%~ 80% of samples (Fig. 2). There was a statistically significant difference in distribution of genotype by year (*χ*^2^ = 141.254, *P* < 0.001).

### Phylogenetic analysis of VP7 genes

**Fig. 3:**
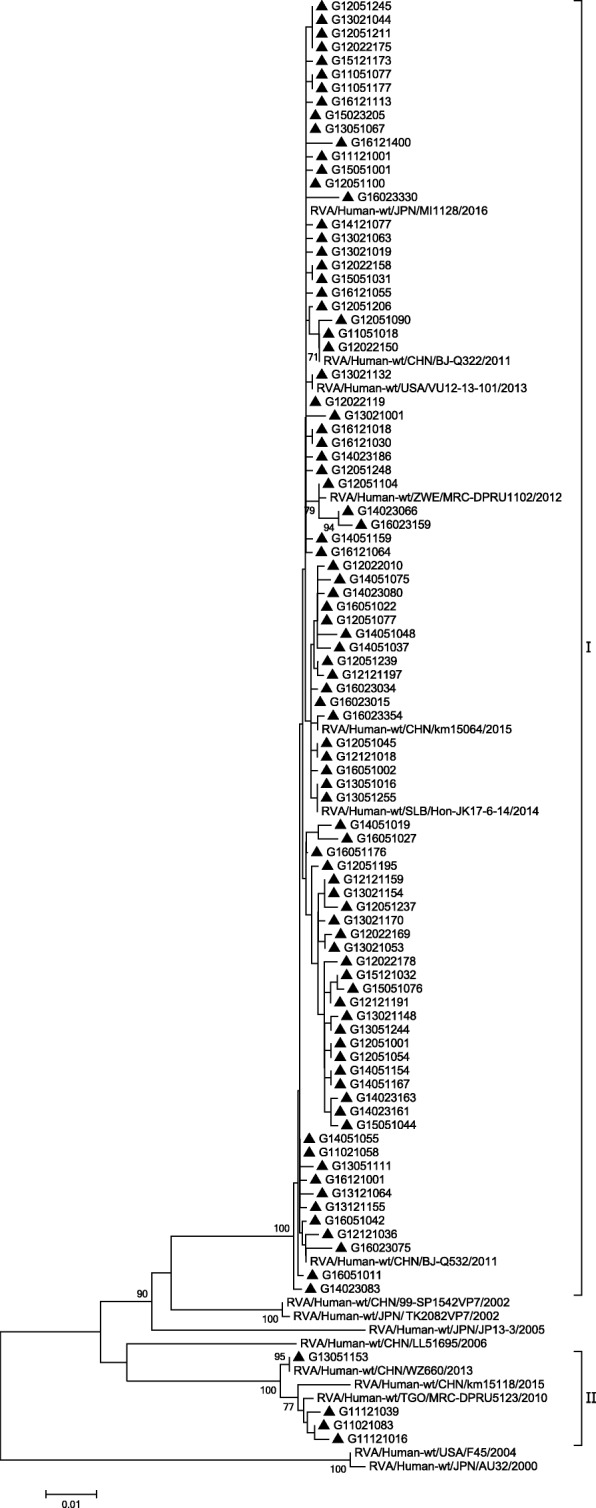
Phylogenetic analysis based on partial VP7 genes (790 bp) of G9 that were identified in Beijing. The trees were generated using the neighbor-joining method with the Kimura 2-parameter model. Bootstrap values estimated with 1000 replicate data sets were indicated at each node. The scale bar indicated the number of nucleotide substitutions per site. Bootstrap values lower than 70% are not shown

**Fig. 4:**
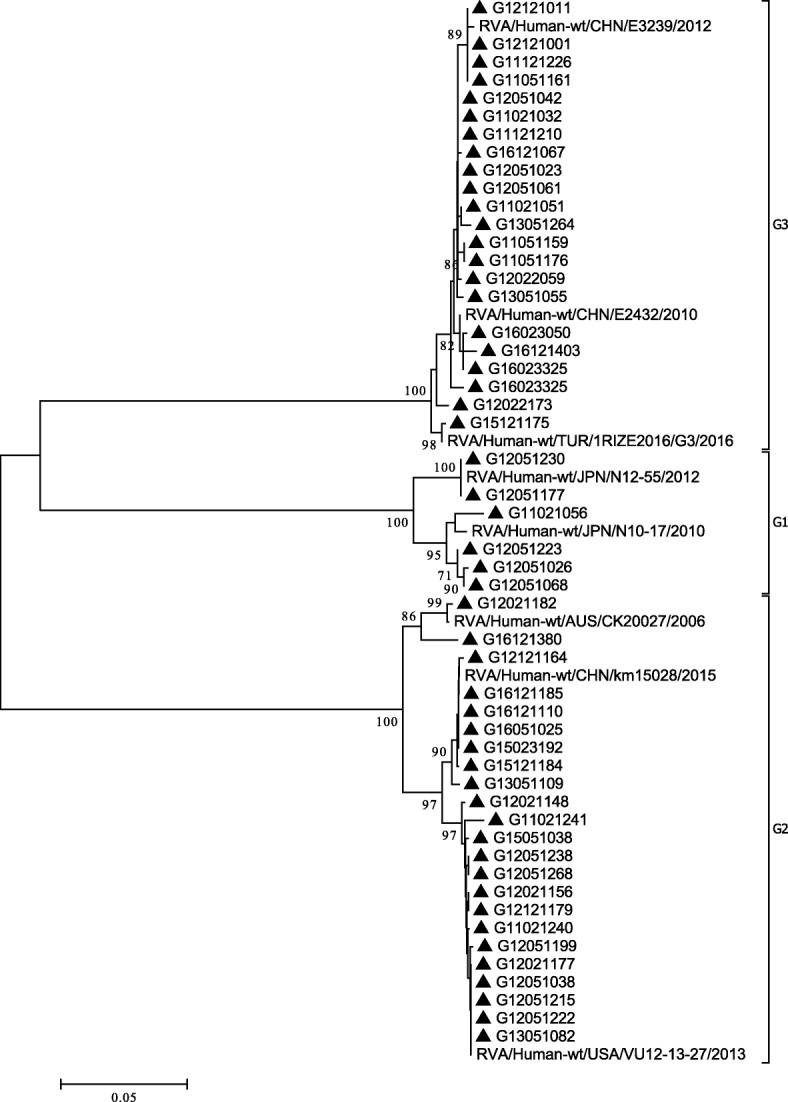
Phylogenetic analysis based on partial VP7 genes (790 bp) of G1, G2, and G3 that were identified in Beijing. The trees were generated using the neighbor-joining method with the Kimura 2-parameter model. Bootstrap values estimated with 1000 replicate data sets were indicated at each node. The scale bar indicated the number of nucleotide substitutions per site. Bootstrap values lower than 70% are not shown

G genotypes were identified by phylogenetic analysis of VP7 genes. In this study, the G9 strains isolated were clustered into two minor lineages (I and II) (Fig. 3). Most of the G9 strains were in lineage I, which exhibited the highest nucleotide similarity (99.1%~ 100.0%) to the strain found in Japan (MI1128). In lineage II, only three strains in 2011 and one strain in 2013 were clustered separately. Lineage II exhibited the highest nucleotide similarity (98.8%~ 100.0%) to the strain found in China (WZ660). Moreover, the phylogenetic analysis of VP7 genes indicated that strains were all clustered correctly for genotypes G1, G2 and G3 (Fig. 4).

### Phylogenetic analysis of VP4 genes

**Fig. 5:**
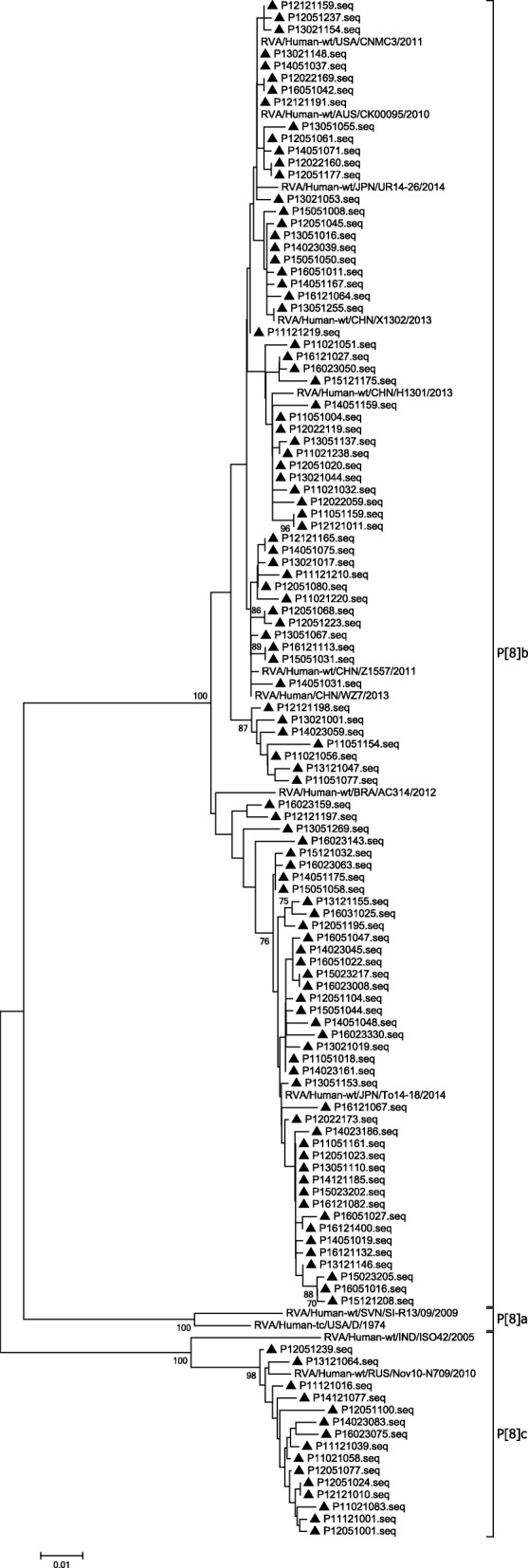
Phylogenetic analysis based on partial VP4 genes (615 bp) of P [8] that were identified in Beijing. The trees were generated using the neighbor-joining method with the Kimura 2-parameter model. Bootstrap values estimated with 1000 replicate data sets were indicated at each node. The scale bar indicated the number of nucleotide substitutions per site. Bootstrap values lower than 70% are not shown

P genotypes were identified by phylogenetic analysis of VP4 genes. In this study, the P [8] strains isolated were clustered into two minor lineages (P[8]b and P[8]c) (Fig. 5). Most of the P [8] strains were P[8]b, which exhibited the highest nucleotide similarity (97.5%~ 100.0%) to the strain found in Australia (CK00095). Fifteen strains in 2011 and 2012 were clustered separately, which exhibited 97.8%~ 99.5% nucleotide similarity to the strain found in Russia (Nov10-N709).

**Fig. 6:**
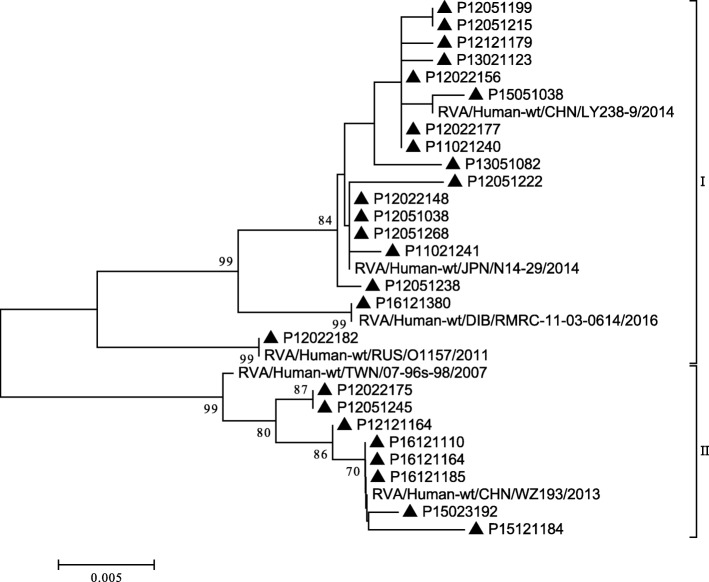
Phylogenetic analysis based on partial VP4 genes (615 bp) of P [4] that were identified in Beijing. The trees were generated using the neighbor-joining method with the Kimura 2-parameter model. Bootstrap values estimated with 1000 replicate data sets were indicated at each node. The scale bar indicated the number of nucleotide substitutions per site. Bootstrap values lower than 70% are not shown

The P [4] strains isolated were clustered into two minor lineages (I and II), similar to the results of Than VT et al. [29]. Phylogenetic analysis of P [4] genotypes in South Korea from 1989 to 2009 revealed that those strains clustered into two lineages, respectively. Lineage I of P [4] genotypes in our study exhibited 97.8%~ 100.0% nucleotide sequence similarity to the strain found in Dibrugarh (RMRC-11-03-0614). Only eight strains of P [4] strains were in lineage II, exhibited 99.3%~ 100.0% nucleotide sequence similarity to the strain found in China (WZ193) (Fig. 6).

## Discussion

Understanding the distribution and antigenic variation of rotavirus infection among children under 5 years old in China can inform vaccination policy in the future. Our study showed that rotavirus was responsible for 20.8% of diarrhea in outpatient children in Beijing, ranging from 16.4 to 27.3%. Children aged 1–2 years old had the highest rotavirus-positive rate (29.0%), followed by those aged 6–11 months old (24.0%), and children aged 2–3 years old (17.9%). The circulation of rotavirus was seasonal, peaking from November to January each year in Beijing. Urban children had higher rotavirus-positive rates compared with children who lived in rural areas, probably reflecting crowding and sanitation. The data also shows significantly different rotavirus-positive rates in different years. G9P [8] had become the dominant genotype in recent years. Some lineages of VP7 and VP4 gene sequences were discovered in previous years, but not in recent years.

Previous studies showed that, the rotavirus-positive rate varied among different regions and years in China. These studies showed that the rotavirus-positive rates of outpatient service ranged from 14.1 to 40.8%, with the rotavirus-positive of hospitalization was even higher in previous studies [30–33]. Our rate was lower than in Gansu Province (66/229, 28.8%) [26], but higher than Shanghai (138/1479, 9.3%) [25].

Most children infected with rotavirus at least once before the age of 5 years old [1, 34]. A previous study [28] also presented similar results which showed children aged 6 months to 3 years old were the most vulnerable to rotavirus infection. Rotavirus-positive rate of children under 6 months is lower (9.0%), possibly due to maternal antibodies remained and less contact with the outside world.

There was a strong association between rotavirus infection in childhood and climate factors, with highest rotavirus-positive rates during the cold season [35]. For every 1 °C increase in mean temperature, the rotavirus incidence reduced by 10% [36]. Data from 11 sentinel hospitals in China from August 2003 through July 2007 [23] showed rotavirus-positive peaks in winter in all regions. Our result was consistent with this finding.

Our research found that children who lived in rural areas had lower rotavirus-positive rates compared with urban children. This result was similar to a study in Northern Cameroon [37]. Less crowding and better sanitation in rural areas may explain this difference. However, medical consultations were the highest in Beijing hospitals, two of which were in the city, one in the suburbs. This might result in sampling bias. Further, children in rural areas may have less access to hospitals or to clinics.

Epidemic strains vary over time and place in both developed and developing countries. G12P [8] was the dominant genotype in children in the United States during 2011 to 2013 [38]. G1P [8] was the most predominant genotype in Japan during 2014 to 2015 [39]. However, G12P [8], G1P [8] and G12P [6] were the most common strains in Myanmar, Sir Lanka and Nepal respectively of south-east Asian from 2009 to 2015 [40]. In China, from 2000 through 2013, G3P [8] was the major stain in Wuhan, and there was genetic evolution [14]. In our study, G3P [8] and G9P [8] were both the dominant genotypes, accounting for 24.2 (24/99) and 22.2% (22/99) respectively in 2011. However, G9 became the main genotype through these years in China [41], accounting for 56.1% (101/180) in Beijing in 2012. Our results confirmed that G9P [8] was the dominant genotype from 2013 to 2016, accounting for more than 70% of samples. Many factors could influence the rotavirus-positive rate in surveillance studies, such as socioeconomic factors [42], access to health facilities, sampling, serotype [43], density of population, vaccination rate [3], crowding, education [44], malnutrition [45], sanitation, geographic and behavioral factors [35]. Our longstanding hospital-based surveillance provided insight into trends in rotavirus infection. Some uncommon strains such as G8P [8], G2P [8], G3P [4] and G9P [4] were detected in this study, but not G12 [46, 47].

Evolutionary trees in this study were used to verify that the G and P strains were divided into different genotypes correctly. There were at least ten branches of VP7 gene sequences of G9 type, originating from people or pigs. Our study showed that G9 strains clustered into two lineages. The main lineage of G9 strains were similar to that in Japan, the United States, China and other countries. In another lineage, there were only four strains. These were similar to strains found in USA (F45) in the past. But in recent 3 years, this strain has not been circulating. The VP4 gene sequences of P [8] clustered into most P[8]b and P[8]c lineages. Only fifteen strains were P[8]c mostly before 2014, similar to the strain found in Russia (Nov10-N709). However, P[8]b was widely prevalent in Beijing in recent years. The P[8]b in Beijing was similar to that in Japan (UR14–26, To14–18), the United States (CNMC3), China (X1302, Z1557) and other countries of the world in recent years. According to previous studies [48, 49], some P[8]b strains might have occurred through reassortment between P[8]a strains and unidentified strains with the P[8]b-VP4 gene. However, P[8]a were not detected in our study.

In conclusion, our study highlights that rotavirus causes a high disease burden in children under 5 years in Beijing, with a change in predominant strains over time. At present, G9P [8] is the dominant strain. Further research is needed to explore the related factors influencing genotypes selection, and correlation between genotypes with clinical illness severity in children. The economic burden of rotavirus-positive and the cost-effectiveness of a vaccination program are also should be considered. Currently there is no specific drug for the rotavirus diarrhea, but vaccines are available and can protect children from illness and death caused by rotavirus. In 2009, WHO recommended that all countries should promote the rotavirus vaccine [50]. RotaTeq® (RV5) and Rotarix® (RV1) were included in the national immunization program in 84 countries by the end of 2015 [4], and 33 African countries by 2016 [51], but not in China. Understanding the local and national distribution of rotavirus strains is vital for targeted research and development of vaccine for China. Available rotavirus vaccination could be considered in China to prevent rotavirus infection and reduce the burden of disease [52, 53].

## Conclusions

Rotavirus was a major cause of childhood diarrhea in Beijing during 2011 to 2016. The rotavirus-positive rate of 2012 and 2013 were higher than 2011, the dominant genotype changed from G3P [8] to G9P [8] in that 2 years. At present, G9P [8] is the dominant strain. Targeted measures, such as available rotavirus vaccination could be considered in China to prevent rotavirus infection and reduce the morbidity and mortality.
